# Supplementary material for: Long-term spatial dynamics of jaguars in a high-density population
Source: PLoS One. 2025 Oct 7;20(10):e0332070. doi: 10.1371/journal.pone.0332070 (PMC12503326; doi:10.1371/journal.pone.0332070)
Supplement: S1 Fig — (PDF) [file pone.0332070.s001.pdf]

S1 Fig Annual activity centres for 11 male jaguars with ≥ 5 years of activity centres (black circles, labelled by year), location of camera traps shown as grey circles; maximum distance between annual activity centres shown as dashed line; graph indicates change in the distance between consecutive annual activity centres through time; ID of individual shown in graph.

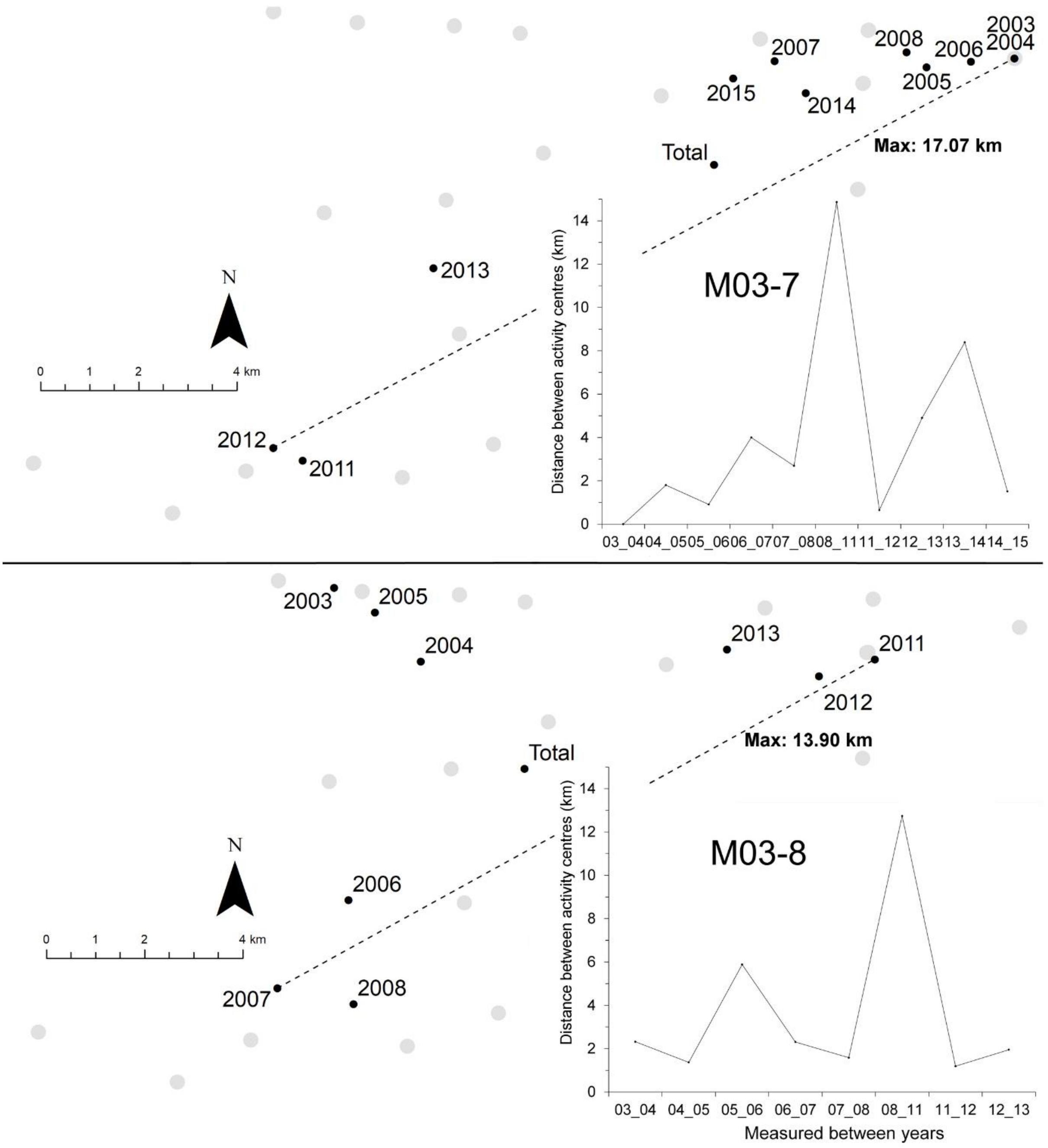

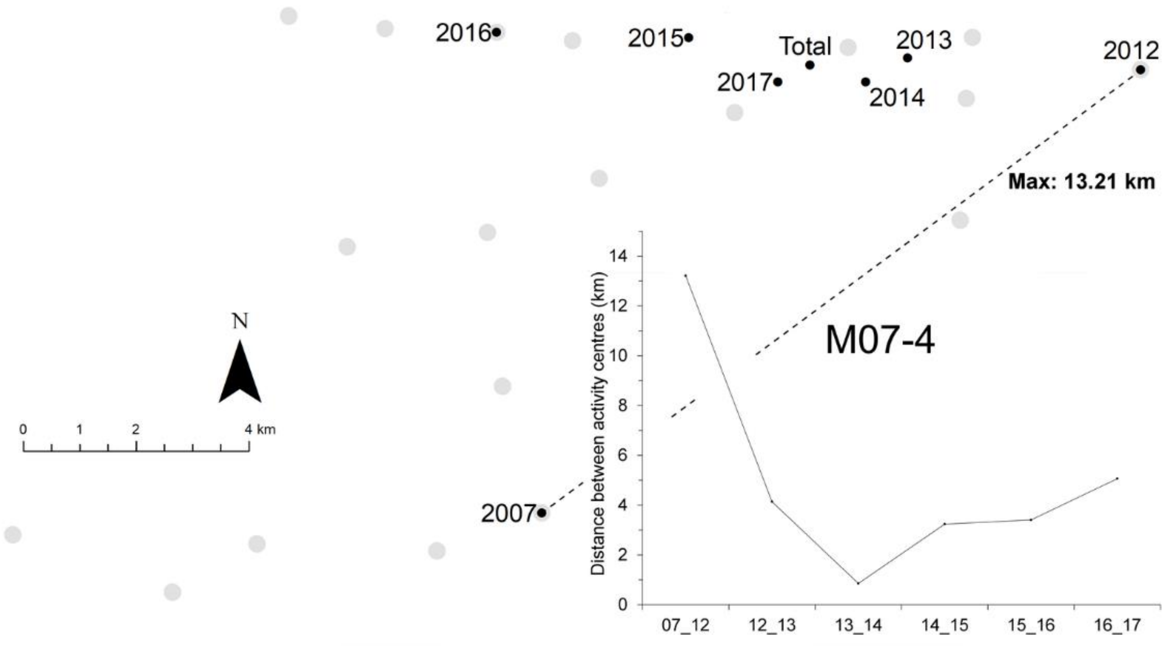
2016
2015
Total
2013
2012
2017
2014
Max: 13.21 km
N
0 1 2 4 km
2007
M07-4
Distance between activity centres (km)
0 2 4 6 8 10 12 14
07_12 12_13 13_14 14_15 15_16 16_17

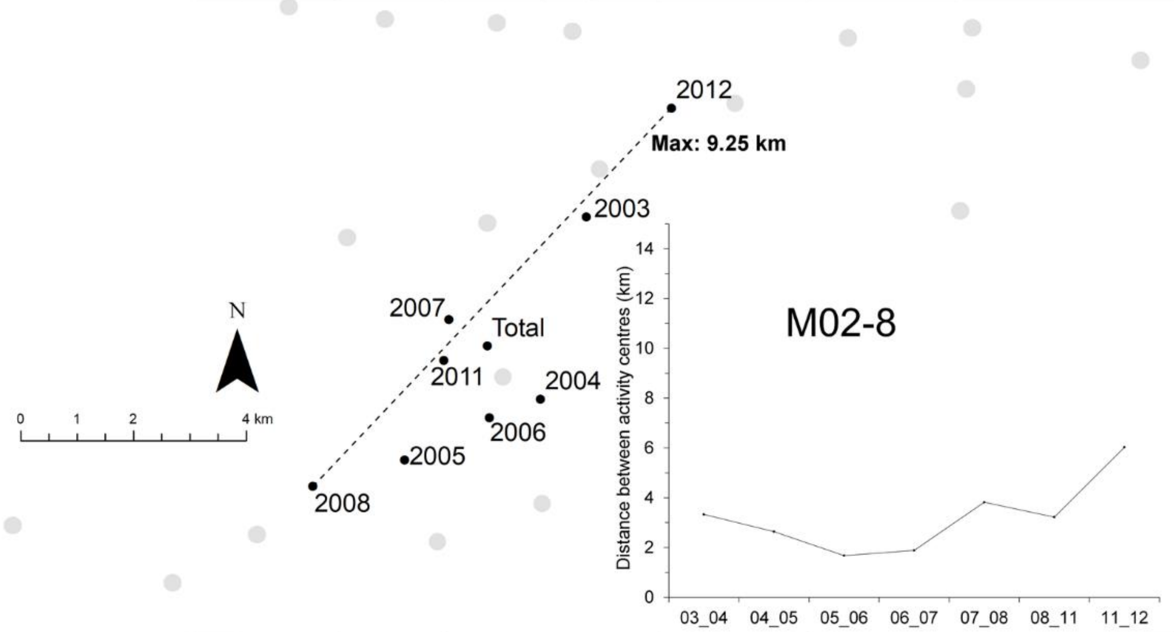
2012
Max: 9.25 km
2003
2007
Total
2011
2004
2006
2005
2008
N
0 1 2 4 km
M02-8
Distance between activity centres (km)
0 2 4 6 8 10 12 14
03_04 04_05 05_06 06_07 07_08 08_11 11_12

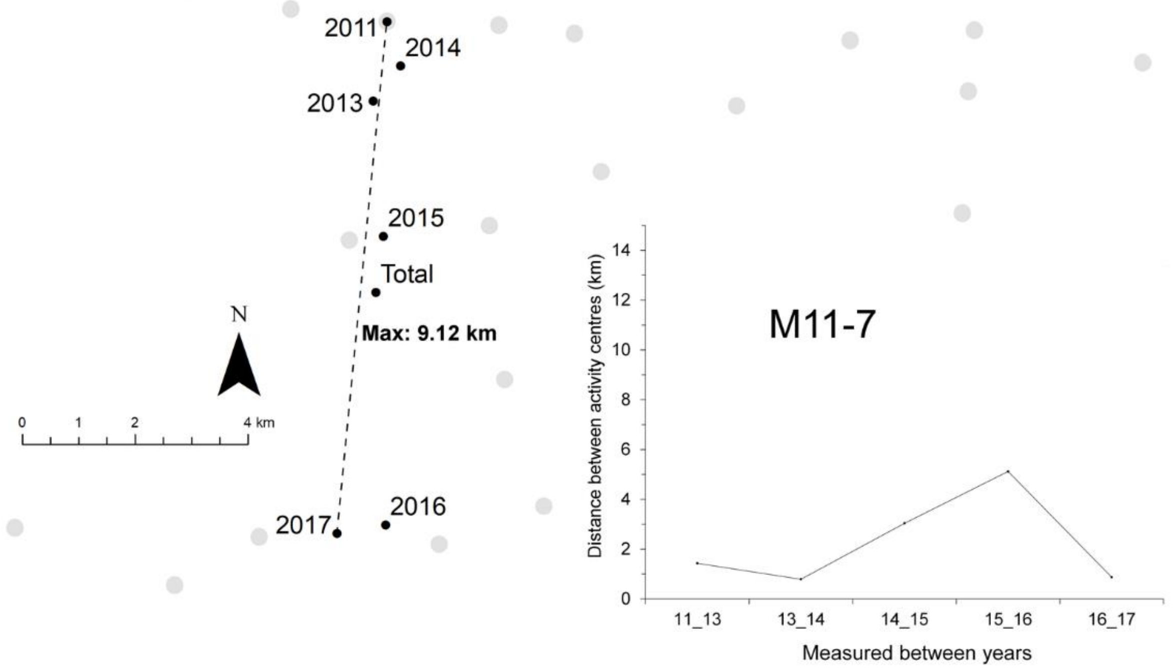
2011
2014
2013
2015
Total
Max: 9.12 km
N
0 1 2 4 km
2017
2016
M11-7
Distance between activity centres (km)
0 2 4 6 8 10 12 14
11_13 13_14 14_15 15_16 16_17
Measured between years

7

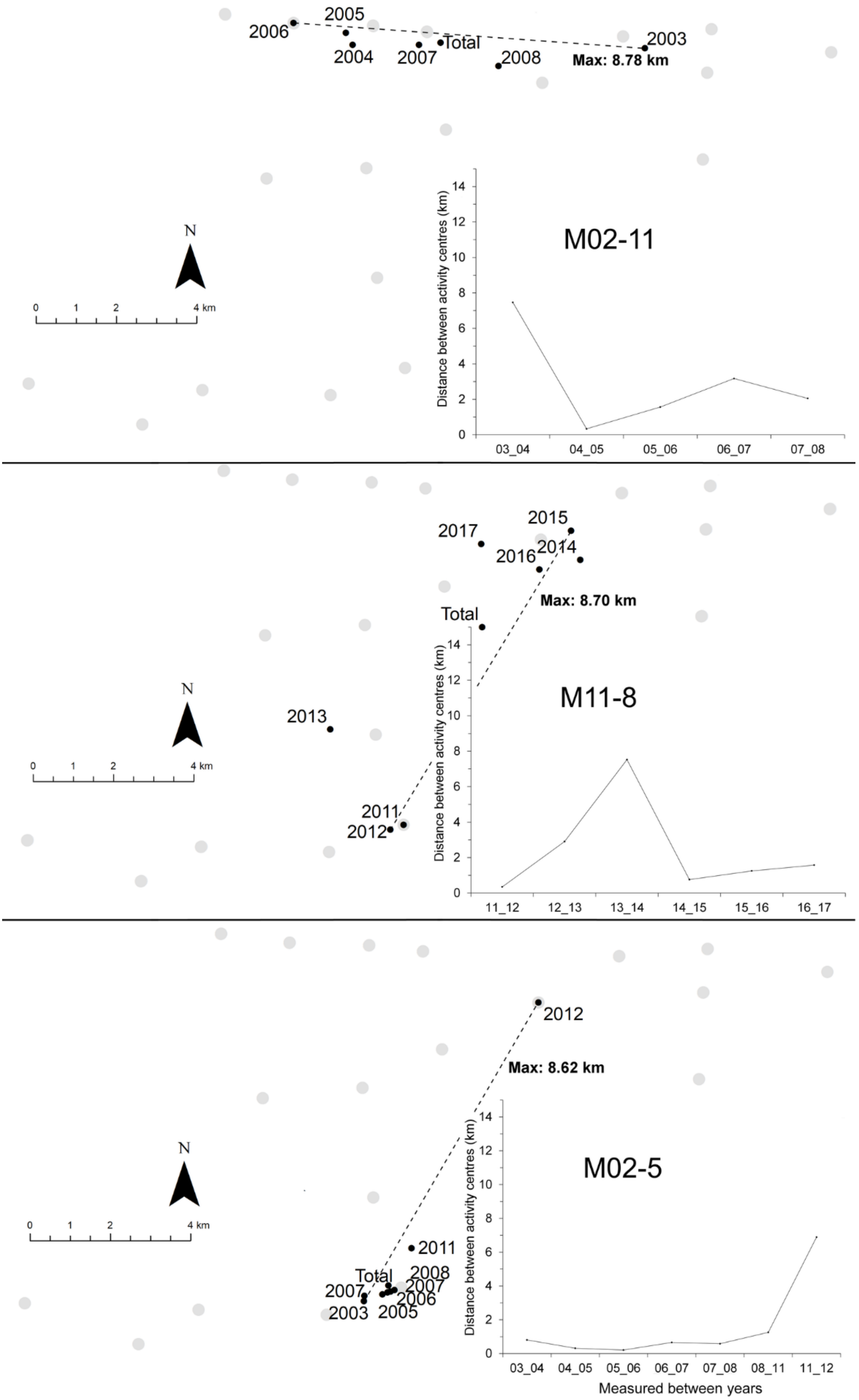
2006
2005
2004
2007
Total
2008
2003
Max: 8.78 km
N
0 1 2 4 km
M02-11
Distance between activity centres (km)
14
12
10
8
6
4
2
0
03_04
04_05
05_06
06_07
07_08
2015
2017
2016
2014
Max: 8.70 km
Total
M11-8
2013
2011
2012
11_12
12_13
13_14
14_15
15_16
16_17
2012
Max: 8.62 km
M02-5
2011
Total
2008
2007
2007
2006
2003
2005
03_04
04_05
05_06
06_07
07_08
08_11
11_12
Measured between years

8

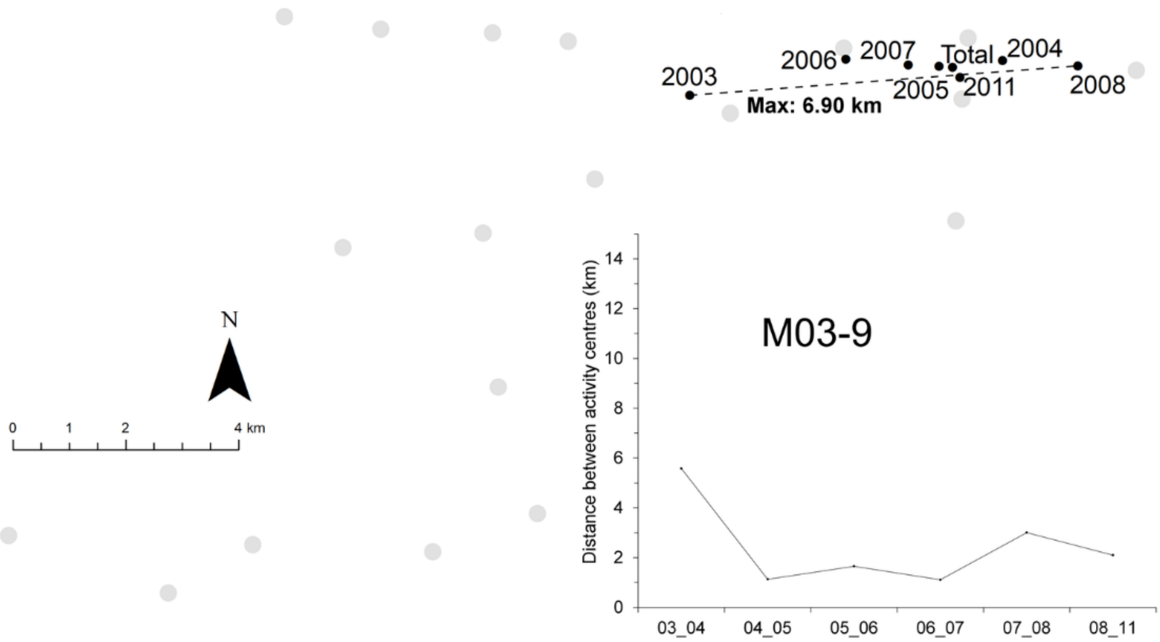

2003
2006
2007
Total
2004
2005
2011
2008
Max: 6.90 km
N
0 1 2 4 km
M03-9
Distance between activity centres (km)
0 2 4 6 8 10 12 14
03_04 04_05 05_06 06_07 07_08 08_11

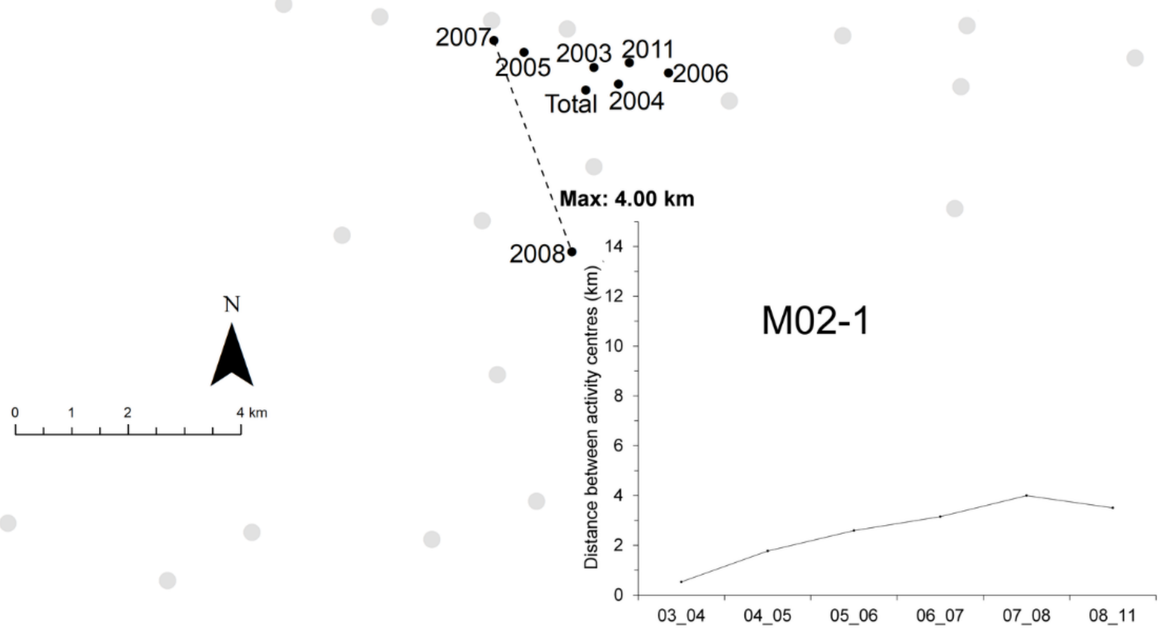

2007
2005
2003
2011
2006
Total
2004
Max: 4.00 km
2008
N
0 1 2 4 km
M02-1
Distance between activity centres (km)
0 2 4 6 8 10 12 14
03_04 04_05 05_06 06_07 07_08 08_11

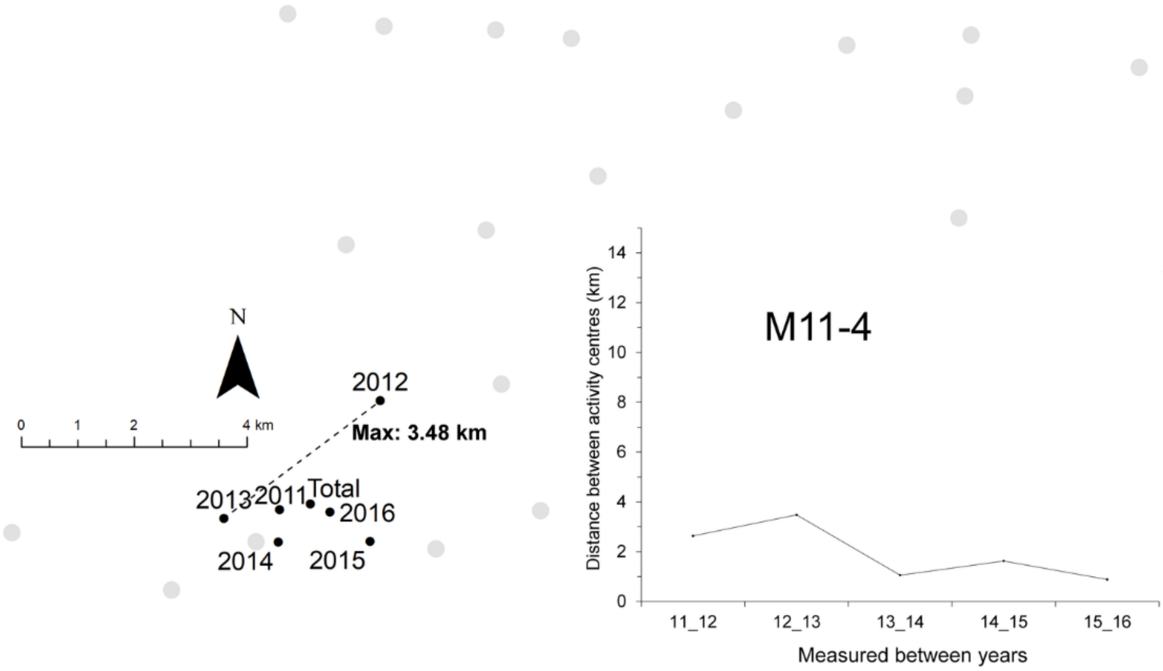

N
0 1 2 4 km
2012
Max: 3.48 km
2013
2011
Total
2016
2014
2015
M11-4
Distance between activity centres (km)
0 2 4 6 8 10 12 14
11_12 12_13 13_14 14_15 15_16
Measured between years
