# Supplementary material for: Long-term spatial dynamics of jaguars in a high-density population
Source: PLoS One. 2025 Oct 7;20(10):e0332070. doi: 10.1371/journal.pone.0332070 (PMC12503326; doi:10.1371/journal.pone.0332070)
Supplement: S2 Fig — (PDF) [file pone.0332070.s002.pdf]

S2 Fig Annual locations of activity centres for male jaguars with ≥ 5 detections per year (black), for the years 2003 to 2008 and 2011 to 2017. The sample size of detections, making up the activity centres, is indicated by the size of the black circle (for legend see year 2003); main survey camera stations shown as grey circles.

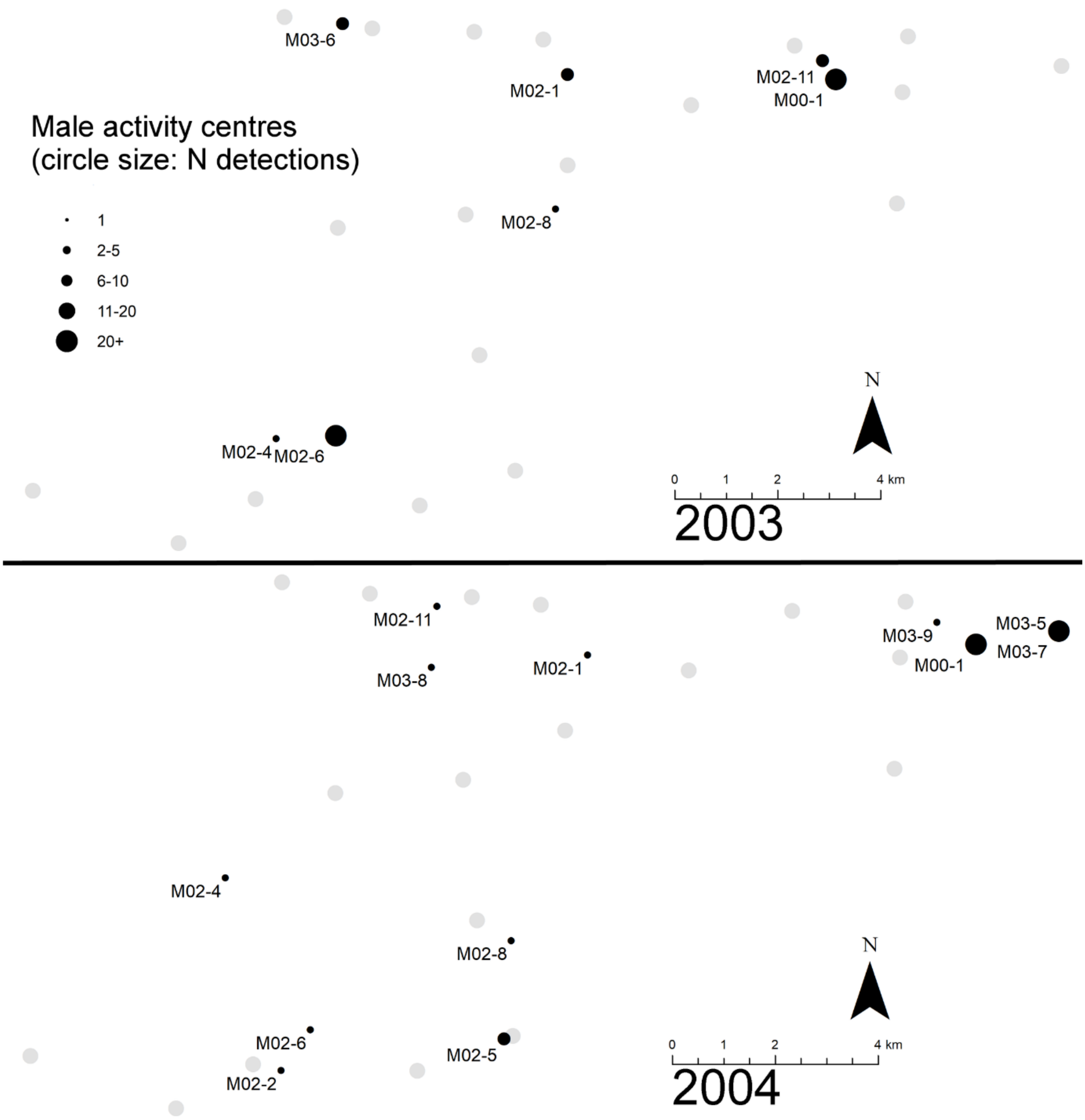

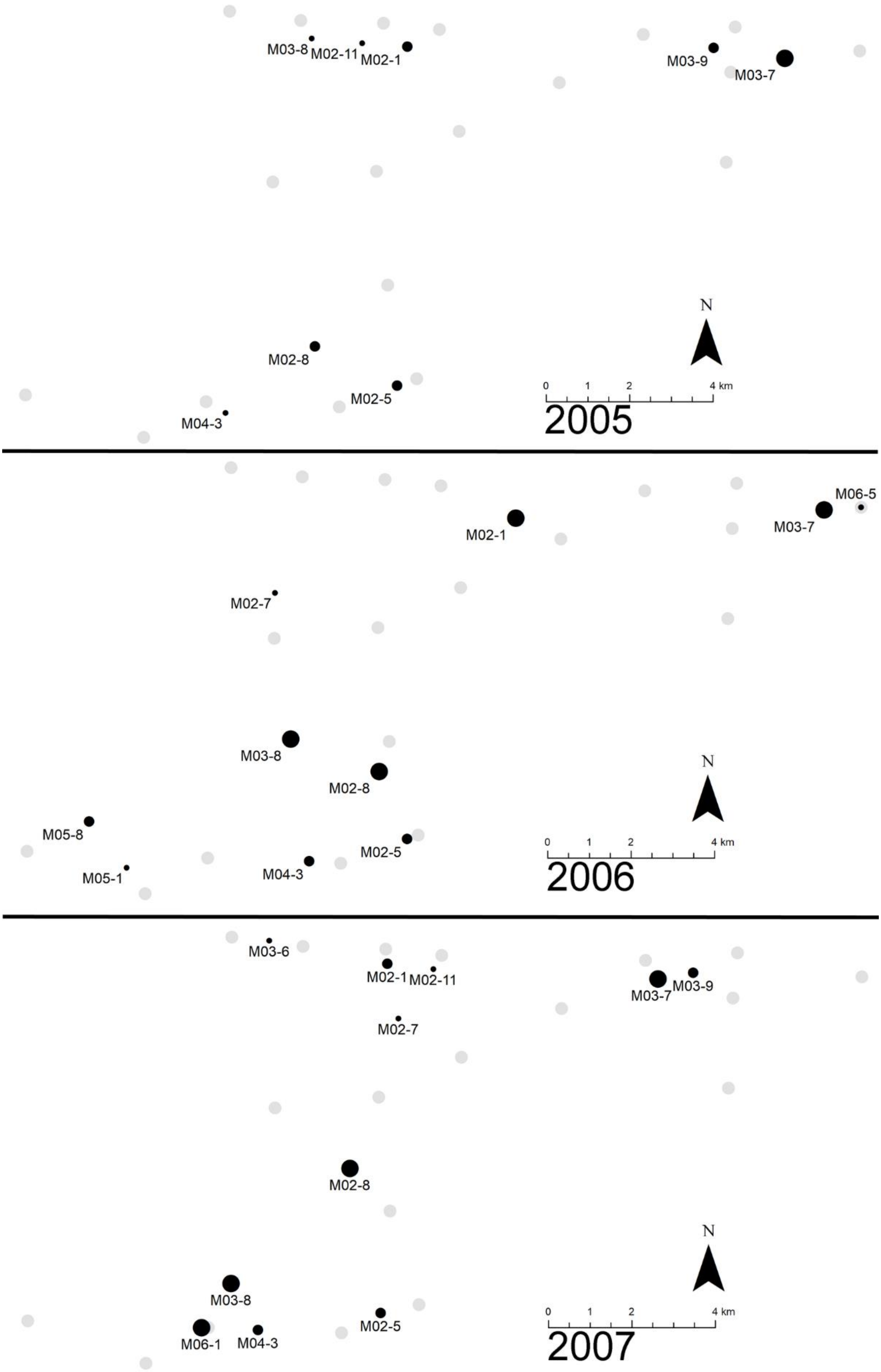
M03-8
M02-11
M02-1
M03-9
M03-7
M02-8
M02-5
M04-3
N
0 1 2 4 km
2005
M06-5
M03-7
M02-1
M02-7
M03-8
M02-8
M05-8
M02-5
M05-1
M04-3
N
0 1 2 4 km
2006
M03-6
M02-1
M02-11
M03-7
M03-9
M02-7
M02-8
M03-8
M02-5
M06-1
M04-3
N
0 1 2 4 km
2007

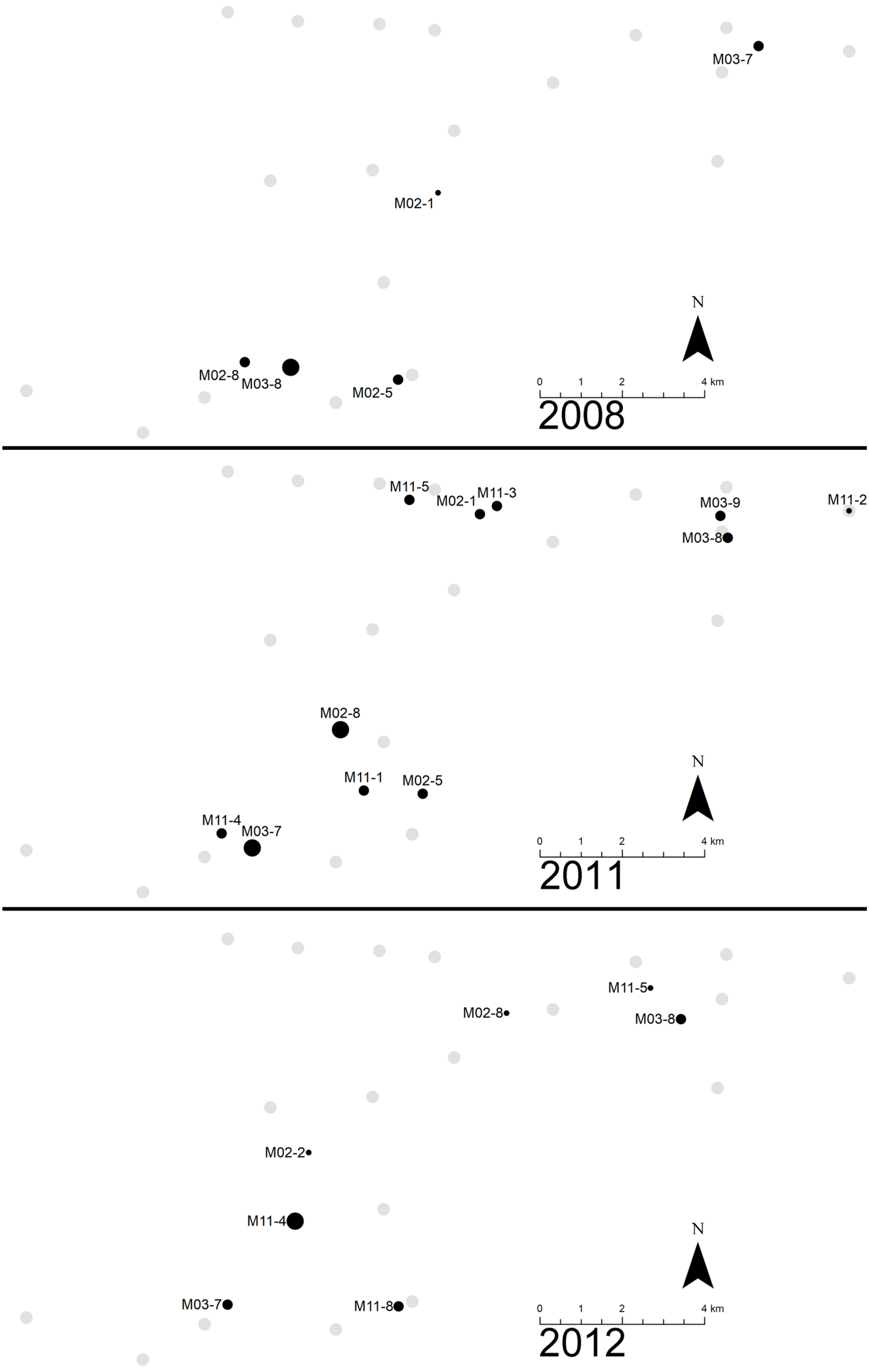
M03-7
M02-1
N
M02-8
M03-8
M02-5
0 1 2 4 km
2008
M11-5
M02-1
M11-3
M03-9
M11-2
M03-8
M02-8
N
M11-1
M02-5
M11-4
M03-7
0 1 2 4 km
2011
M11-5
M02-8
M03-8
M02-2
M11-4
N
M03-7
M11-8
0 1 2 4 km
2012

13

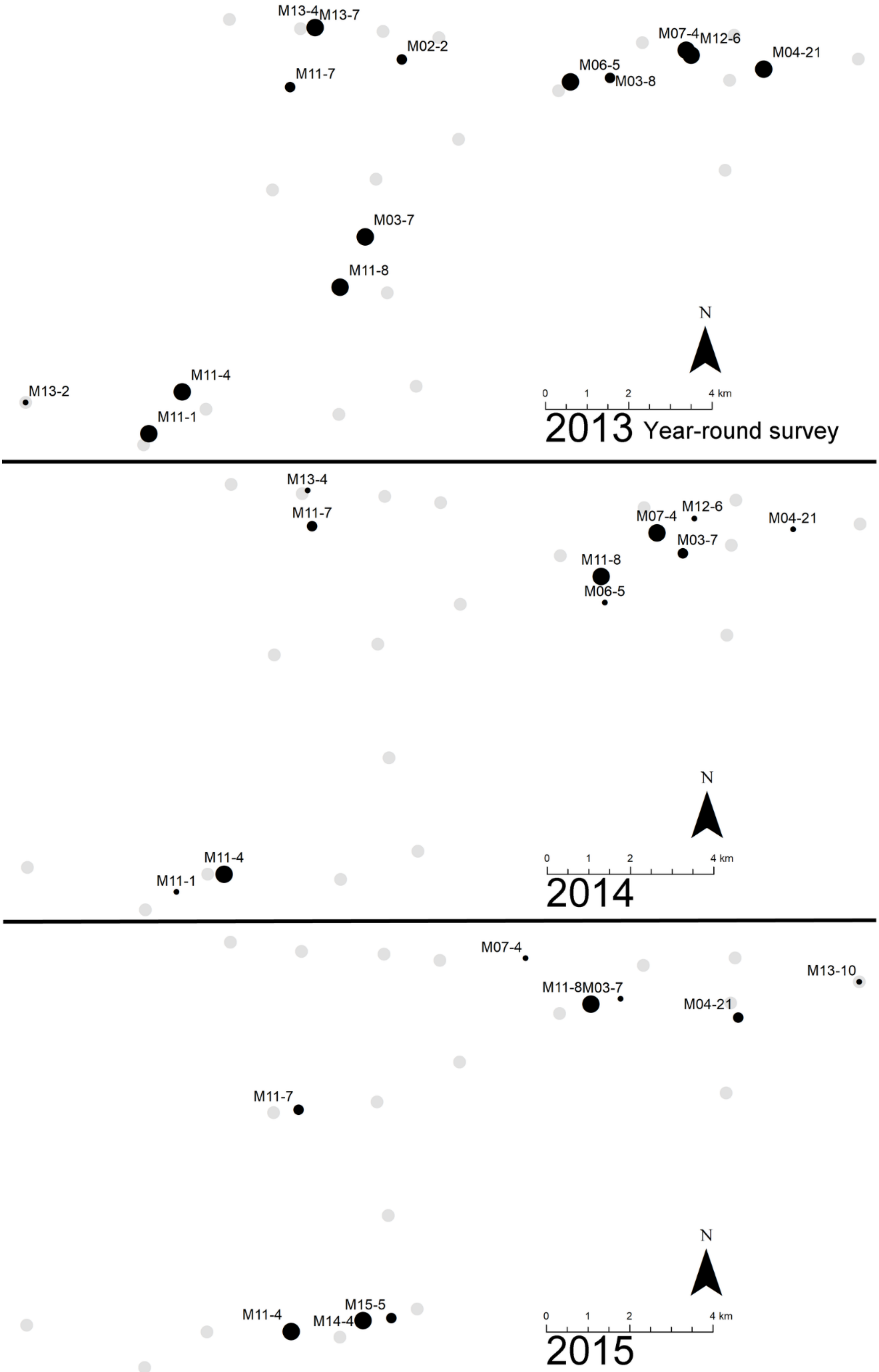
M13-4
M13-7
M02-2
M11-7
M07-4
M12-6
M04-21
M06-5
M03-8
M03-7
M11-8
N
0 1 2 4 km
M13-2
M11-4
M11-1
2013 Year-round survey
M13-4
M11-7
M12-6
M07-4
M04-21
M03-7
M11-8
M06-5
N
0 1 2 4 km
M11-4
M11-1
2014
M07-4
M13-10
M11-8
M03-7
M04-21
M11-7
N
0 1 2 4 km
M15-5
M11-4
M14-4
2015

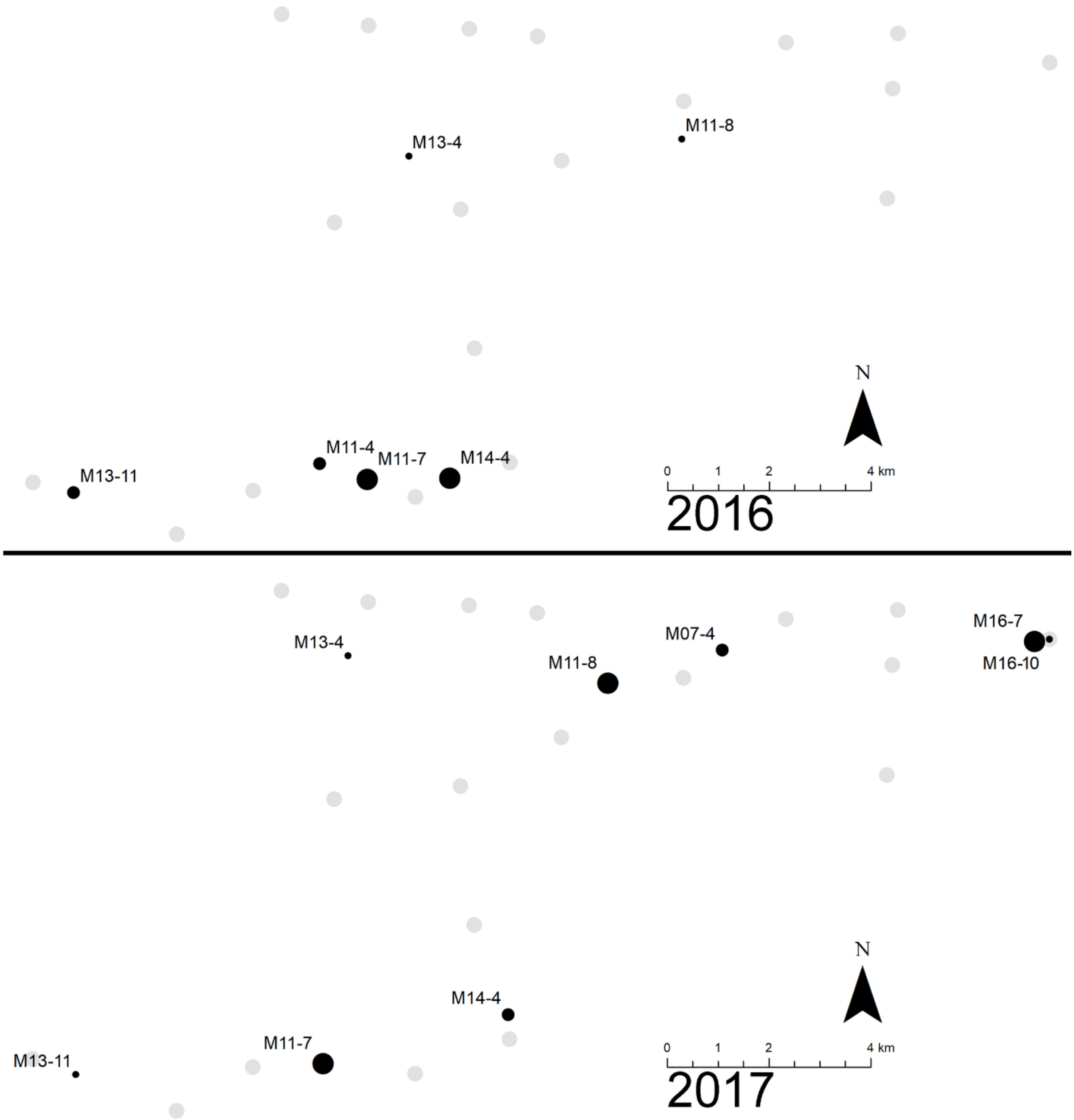
M13-4
M11-8
N
M11-4
M11-7
M14-4
M13-11
0
1
2
4 km
2016
M13-4
M07-4
M16-7
M11-8
M16-10
N
M14-4
M11-7
M13-11
0
1
2
4 km
2017
