# Supplementary material for: Long-term spatial dynamics of jaguars in a high-density population
Source: PLoS One. 2025 Oct 7;20(10):e0332070. doi: 10.1371/journal.pone.0332070 (PMC12503326; doi:10.1371/journal.pone.0332070)
Supplement: S1 File — (PDF) [file pone.0332070.s003.pdf]

Supplementary Methods

To assess whether the distance between pairs of activity centres were a valid means of analysing overlap, we also calculated the volume of intersect between yearly home-range kernels (95% KHR) and tested for a correlation between the two measures of overlap. For each jaguar with at least 7 detections from at least five locations per year (minimum requirement for this analysis: locations: mean = 6.6, SD = 1.9 locations; detections: mean = 24.4, SD = 17.5 detections), we calculated the annual KHR, and used the KHRs to calculate the volume of intersect (VI) index, estimating home range overlap within individuals across years, and between pairs of individuals within years, (1), using the adehabitatHR package (2) within r (3). The VI index ranges from 0 (no overlap) to 1 (complete overlap). We defined 'high overlap' as VI ≥ 0.5 (i.e. ≥ 50% of a pair of ranges overlaps).

We calculated Pearson correlations between VI overlap values and distances between activity centres for the subsample of dyad pairs for which we could calculate VI overlap values. We calculated this within individuals across years, and between pairs of different individuals within years.

Shifts in male range use

We assessed the consistency of individuals' ranges through time for all jaguars with >1 annual 95% KHR. To do this, we created a matrix of VI index values for each individual across the years they were detected. High VI (≥ 0.5) suggests that an individual's spatial range remained constant, while low VI (< 0.5) suggests that an individual's range shifted with time.

Overlap and crowding between males

For each individual, for each year that they were detected, we selected the maximum VI recorded with another male. Then we calculated the mean of these maximum VIs for each individual, as a measure of extent to which it overlapped, on average, with the nearest male. To assess the level of crowding experienced by each individual, during each year we summed the number of males with which it had a VI ≥ 0.5, and then averaged this over the years that it was detected.

For individuals with at least five years of kernel data, we assessed the stability of range overlap through time by summing the number of years the individual maintained VI ≥ 0.5 overlap with the same individual.

We detected 23 males with at least 7 detections per year at ≥ 5 locations, allowing us to estimate their annual kernel home ranges, KHR (1 to 8 ranges per individual; mean = 3, SD = 2.3, n = 69) and the volume of intersect (VI) index between every possible pairing. We found a strong significant negative relationship between the VI values and the distances between the equivalent activity centre pairs (Pearson correlation: within individuals between years r = -0.91, p < 0.01, n = 131, between individuals within years r = -0.90, p < 0.01, n = 152)Shorter distances between activity centres reflect larger VIs, with distances ~ 2 km equating to a VI of ~ 0.6. Because of the strong correlation, we felt confident that the distance between pairs of activity centres could be used as a proxy for VI overlap. As estimates of activity centres are less data-hungry than estimates of VI overlap, using activity centres allowed us to make use of data from more individuals than the VI analysis, so boosting our sampling size for analysing male space use.

Below we present the results of male space use based on VI overlap, illustrating similar patterns as those based on activity centres (main manuscript).

Shifts in male range use

We had sufficient data from 16 jaguars to estimate their annual KHRs and overlap (VI) within individuals between years (2 to 8 years per individual). On average, an individual's annual KHRs overlapped < 0.5 between any pair of years (mean overlap between annual KHRs = 0.43, ± SD = 0.26, range 0.23 to 0.86, n = 131 pairwise comparisons) with approximately half (67/131, 51%) of the pairwise comparisons < 0.5, suggesting that home range location changed through time.

Overlap and crowding between males

We had sufficient data to estimate annual KHRs and overlap (VI) between 23 males within years. On average, the maximum extent of overlap of each male with another male equated to approximately half of its detected range (extent of overlap: mean of the maximum VI per individual per year = 0.56, SD = 0.13, n = 69). On average, males shared their detected range to this extent with at least one male each year (level of crowding: mean number of VI values ≥ 0.5 per individual per year = 0.97, SD = 0.68). The highest level of crowding we detected was for three individuals, with each overlapping with four other males ≥ 0.5.

High overlap (VI ≥ 0.5) was not maintained between the same pairs of individuals (dyads) through time. For the seven males for which we could calculate VI values across multiple years (range: 5 to 8 years), most showed high overlap with different individuals across the

years: two overlapped with different males each year, one overlapped with the same male only for two years, while the remaining four males, each being detected for ≥ 7 years, maintained the same dyads for three years.

Relation between area overlap and distance

Using two circular home ranges of 100 km^2, we created a reference table of the relation between the level of overlap between the two home ranges (grey area in Fig SM) and the distance between the centres of the home ranges (arrow in Fig SM). Table SM indicates the area of overlap per 1 km distance increase.

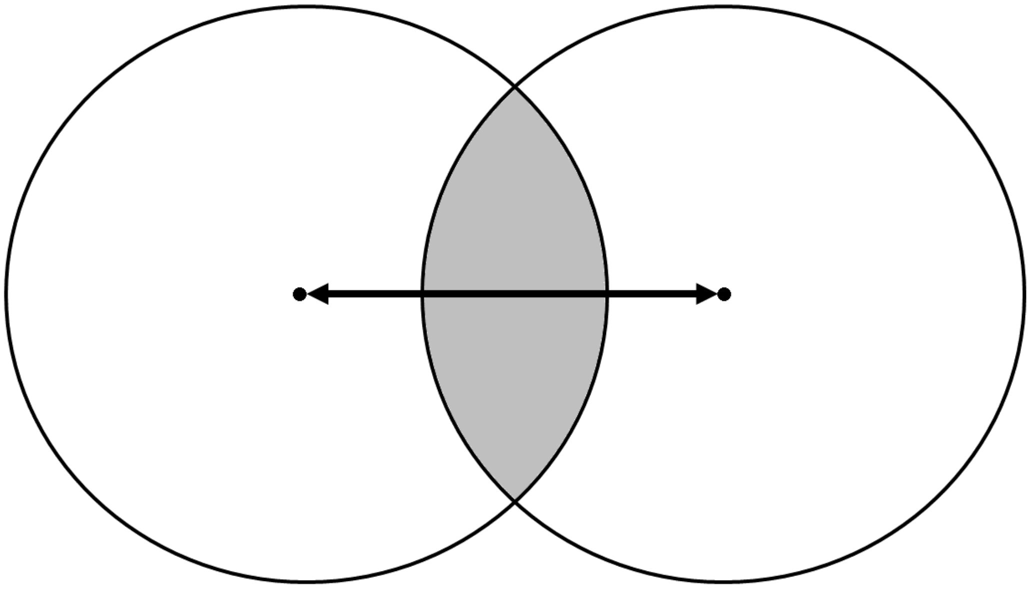

Fig SM. Relation of distance between the centres of two circular 100 km^2 home ranges (indicated by the arrow), and the area of overlap (indicated by the grey area).

Table SM, Relation between the distance between centres of two circular 100 km² home ranges, and area overlap in 500 m increments of distance (see Figure SM for visual representation)

Distance (km)	Area (km²)
0.5	94
1	89
1.5	83
2	78
2.5	72
3	67
3.5	61
4	56
4.5	51
5	45
5.5	40
6	36
6.5	31
7	26
7.5	22
8	18
8.5	14
9	11
9.5	7
10	5

References

1. Fieberg J, Kochanny CO. Research and Management Viewpoint QUANTIFYING HOME-RANGE OVERLAP: THE IMPORTANCE OF THE UTILIZATION DISTRIBUTION. Vol. 69, JOURNAL OF WILDLIFE MANAGEMENT. 2005.
2. Calenge C. The package “adehabitat” for the R software: A tool for the analysis of space and habitat use by animals. Ecol Modell [Internet]. 2006;197(3):516–9. Available from: https://www.sciencedirect.com/science/article/pii/S0304380006001414

3. R Core Team. R: A language and environment for statistical computing. R Foundation for Statistical Computing [Internet]. Vienna: R Foundation for Statistical Computing; 2022. Available from: https://www.r-project.org/
