# Supplementary material for: Long-term spatial dynamics of jaguars in a high-density population
Source: PLoS One. 2025 Oct 7;20(10):e0332070. doi: 10.1371/journal.pone.0332070 (PMC12503326; doi:10.1371/journal.pone.0332070)
Supplement: S2 File — (PDF) [file pone.0332070.s004.pdf]

Supplementary Case Studies

Case 1 (Range take over; see also Harmsen et al 2010): An apparent takeover, similar as described by Rabinowitz and Nottingham (1986), with the disappearance of one established male followed by the range expansion/shift of two neighbouring males. A prime (or older) male, first detected in 2000 in the study area, accounted for the majority of detections in this area until his final detection in August 2004 (Male M00-1, see Fig Case1). Following his disappearance, the number of detections of two males, M03-7 and M03-9, first detected in 2003 at the fringes of this area,, increased, and their detected ranges expanded (S1Fig and Fig Case1). M03-9 remained in this area until 2011, while M03-7 moved away from the area in 2011, and returned in 2014 until he disappeared from our trapping record in 2015 (S1Fig).

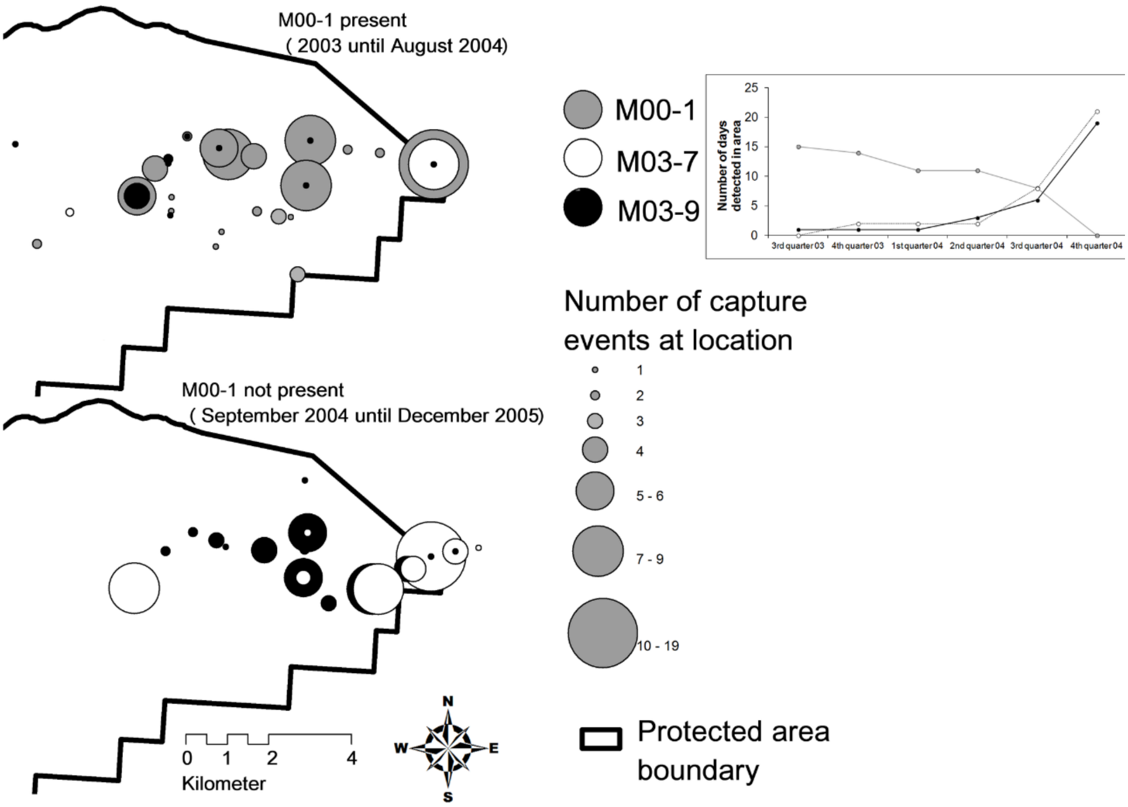

Fig Case 1 Camera trap records for three male jaguars (M00-1(grey), M03-7(white), M03-9 (black)); Upper panel: Detections from 2003 until August 2004, Lower panel: Detections from September 2004 until 2005; Size of the dots indicates number detections per location; The inserted graph shows the overall number of detections for the three males through time.

Case 2 (Repeated close encounters between neighbours): From 2003 through 2006, M03-5, had four consecutive years of high overlap and three consecutive years of close encounters with M03-7.

Case 3 (Repeated close encounters between neighbours): From 2006 through 2011, M02-5 had three years of high overlap and four consecutive years of close encounters with M02-8. In 2012, the activity centres of both males moved approximately 7 km; thereafter they were no longer detected (S1Fig, M02-5 only had two detections in 2012 and thus his activity centre is included based on these two last points of detection).

Case 4 (Repeated close encounters between non-neighbours): A pair of males (M02-1 and M03-8) with low overlap throughout their lives had close encounters every year over five years, and marginal close encounters (24-36h) for the remaining three years until the older individual (M02-1) disappeared from our trapping record (S1Fig, S2Fig).

Case 5 (Repeated close encounters between non-neighbours): M02-8 and M03-8 (same individuals as for case 3 and case 4), showed high frequency of close encounters across 4 years, between 2004 and 2012 (no data for the years 2009 and 2010). For only one of the 4 years, they showed high overlap in activity centres (2008). Both individuals showed no sign of interaction with the other dyad members of case 3 and 4 (M02-8 did not interact with M02-1 and M03-8 did not interact with M02-5).

Case 6 (Sibling association): A tourist visiting the CBWS photographed two male jaguars in 2017, while walking along the entrance road. The two jaguars were close together in visual range and close proximity to one another. The two individuals seemed comfortable with one another, with no sign of antagonism. The two males were both first detected in 2016 (M16-7 and M16-10). The dyad pair had two close encounters with one another and with an older female in 2016 on the same road, suggesting that she was their mother and they were brothers.
