# Supplementary material for: Long-term spatial dynamics of jaguars in a high-density population
Source: PLoS One. 2025 Oct 7;20(10):e0332070. doi: 10.1371/journal.pone.0332070 (PMC12503326; doi:10.1371/journal.pone.0332070)
Supplement: Attachment — Submitted filename: ResponseReviewers_BHarmsen.docx [file pone.0332070.s007.docx]

Dear Editor and reviewers

Many thanks for your thorough and thoughtful reviews. We think the manuscript has highly improved due to your recommendations and below you can find specific replies to your comments with line indications according to the last version without track changes.

Yours sincerely,

Bart J. Harmsen (PhD)

6. Review Comments to the Author

Please use the space provided to explain your answers to the questions above. You may also include additional comments for the author, including concerns about dual publication, research ethics, or publication ethics. (Please upload your review as an attachment if it exceeds 20,000 characters)

Reviewer #5: The manuscript “Long-term spatial dynamics of jaguars in a high-density population” brings us an excellent analysis of the socio-spatial dynamics of a jaguar population in Belize. I consider the manuscript to be very well written and to have a good statistical approach to the objectives described. After reading the article and the comments that had already been made by three other reviewers in rounds prior to my evaluation, my only suggestions would be these:

1) reduce the introduction by 10-20% as it is too long

***We reduced the introduction by ~ 15% and we think it has improved considerably***

2) In this paper, the authors could have considered doing spatiotemporal analysis, not just separating spatial and temporal analysis. Please see, for example, the papers of Karanth et al. (2017), Pereira et al. (2024) and Duarte et al. (2025) to get an idea of a possible spatiotemporal approach that considers both the sites of the cameras and the time at which the photo or video was taken, which would give us an idea of whether there is a temporal exclusion between males and females at each of the sampled site. I understand that this would not be the objective of the present study, and the authors can consider this in a future analysis. However, perhaps the authors could mention this in the discussion, considering it as a future approach that could shed more light on socio-spatial dynamics issues.

***We read and included the suggested references inserting a statement at the end of the manuscript, now reading:***

***Lines 615-621. We recognise the recent development of robust methods to assess spatio-temporal interactions between species using an occupancy framework within a single analysis (e.g. (64–66), and the potential to apply it to studies of interactions between individuals. However, in a study of carnivore sympatry, Karanth et al. (65) noted that the method may be biased with low sample sizes. As such, it may be unsuitable for assessing interactions between individuals unless there are sufficient detections of every individual (across years).***

References
Duarte HO, Rosalino LM, Toledo JJ, Hilário RR, Carvalho WD. 2024. Spatiotemporal interactions between jaguars (Panthera onca) and their potential prey in Amazonian islands. Ecological Research 40(2):217-227. <https://doi.org/10.1111/1440-1703.12522>

Karanth KU, Srivathsa A, Vasudev D, Puri M, Parameshwaran R, Kumar NS. 2017. Spatio-temporal interactions facilitate large carnivore sympatry across a resource gradient. Proceedings of the Royal Society B: Biological Sciences 284(1848):20161860. <https://doi.org/10.1098/rspb.2016.1860>

Pereira R, Matias G, Santos-Reis M, Rosalino LM. 2024. Influence of habitat edges on spatial and spatio-temporal occurrence patterns of mesocarnivores in landscapes dominated by Eucalyptus plantations. Forest Ecology and Management, 572, 122257. <https://doi.org/10.1016/j.foreco.2024.122257>

Many thanks for all your thoughts and help.
